# Enhancing Lung Cancer Care in Portugal: Bridging Gaps for Improved Patient Outcomes

**DOI:** 10.3390/jpm14050446

**Published:** 2024-04-24

**Authors:** Raquel Ramos, Conceição Souto Moura, Mariana Costa, Nuno Jorge Lamas, Renato Correia, Diogo Garcez, José Miguel Pereira, Carlos Sousa, Nuno Vale

**Affiliations:** 1 PerMed Research Group, Center for Health Technology and Services Research (CINTESIS), Rua Doutor Plácido da Costa, 4200-450 Porto, Portugal; raquel_ramos00@hotmail.com (R.R.); carlos.sousa@unilabs.com (C.S.); 2 CINTESIS@RISE, Faculty of Medicine, University of Porto, Alameda Professor Hernâni Monteiro, 4200-319 Porto, Portugal; 3 Molecular Diagnostics Laboratory, Unilabs Portugal, Centro Empresarial Lionesa Porto, Rua Lionesa, 4465-671 Leça do Balio, Portugal; mariana.costa@unilabs.com (M.C.); nuno.lamas@external.unilabs.com (N.J.L.); 4 Pathology Laboratory, Unilabs Portugal, Rua Manuel Pinto de Azevedo 173, 4100-321 Porto, Portugal; conceicao.souto.moura@external.unilabs.com; 5 Anatomic Pathology Service, Pathology Department, Centro Hospitalar Universitário de Santo António (CHUdSA), Largo Professor Abel Salazar, 4099-001 Porto, Portugal; 6 Life and Health Sciences Research Institute (ICVS), School of Medicine, Campus de Gualtar, University of Minho, Rua da Universidade, 4710-057 Braga, Portugal; 7 Technology & Innovation Department, Unilabs Portugal, Rua Manuel Pinto de Azevedo 173, 4100-321 Porto, Portugal; renato.correia@unilabs.com (R.C.); diogo.garcez@unilabs.com (D.G.); 8 Radiology Department, Unilabs Portugal, Rua de Diogo Botelho 485, 4150-255 Porto, Portugal; josemiguelpj@gmail.com; 9 Department of Community Medicine, Health Information and Decision (MEDCIDS), Faculty of Medicine, University of Porto, Rua Doutor Plácido da Costa, 4200-450 Porto, Portugal

**Keywords:** lung cancer, patient journey, gaps, clinical management, life quality, survival

## Abstract

Lung cancer has the highest incidence and cancer-related mortality worldwide. In Portugal, it ranks as the fourth most common cancer, with nearly 6000 new cases being diagnosed every year. Lung cancer is the main cause of cancer-related death among males and the third cause of cancer-related death in females. Despite the globally accepted guidelines and recommendations for what would be the ideal path for a lung cancer patient, several challenges occur in real clinical management across the world. The recommendations emphasize the importance of adequate screening of high-risk individuals, a precise tumour biopsy, and an accurate final diagnosis to confirm the neoplastic nature of the nodule. A detailed histological classification of the lung tumour type and a comprehensive molecular characterization are of utmost importance for the selection of an efficacious and patient-directed therapeutic approach. However, in the context of the Portuguese clinical organization and the national healthcare system, there are still several gaps in the ideal pathway for a lung cancer patient, involving aspects ranging from the absence of a national lung cancer screening programme through difficulties in histological diagnosis and molecular characterization to challenges in therapeutic approaches. In this manuscript, we address the most relevant weaknesses, presenting several proposals for potential solutions to improve the management of lung cancer patients, helping to decisively improve their overall survival and quality of life.

## 1. Introduction

**Figure 1:**
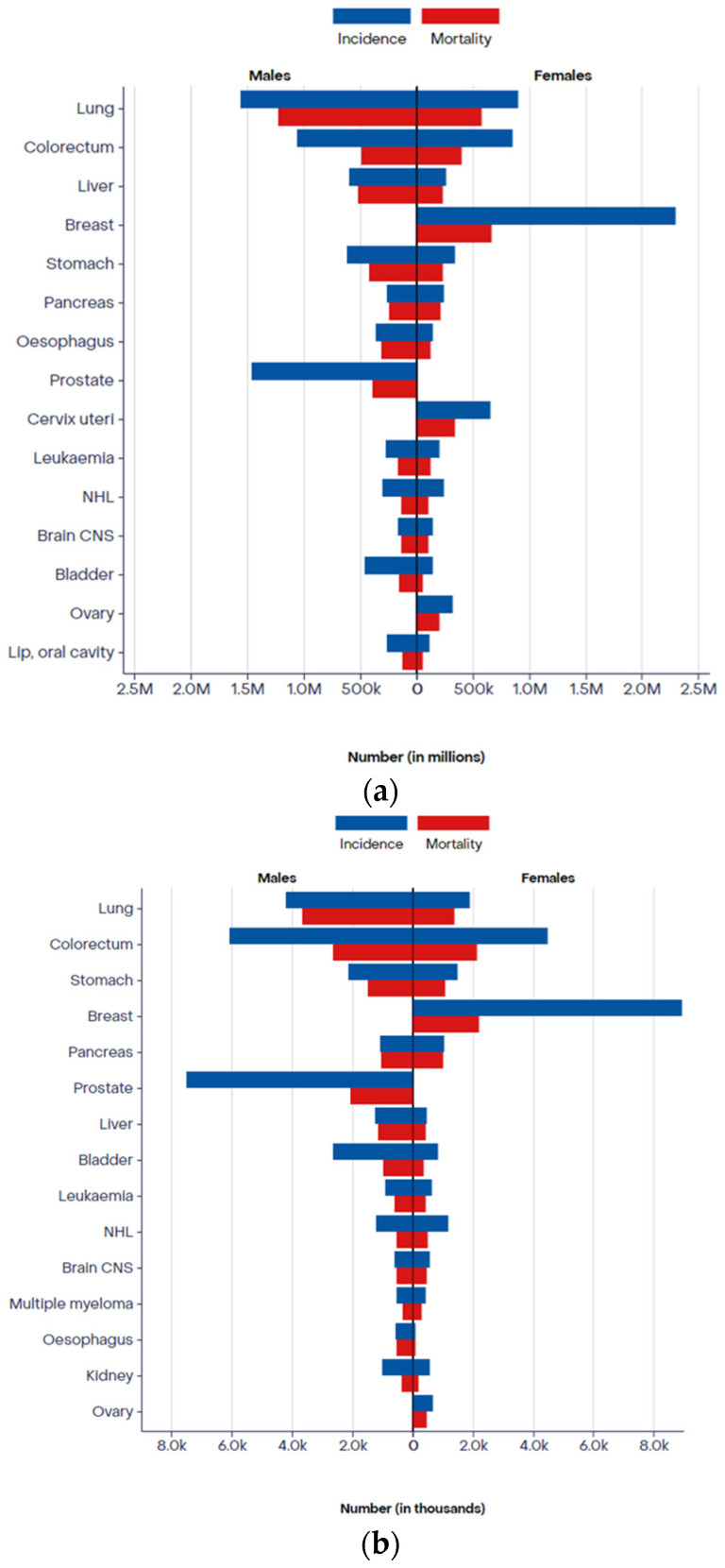
Incidence and mortality of the top 15 cancer sites in males and females (**a**) worldwide (**b**) and in Portugal. Available online: https://gco.iarc.fr/ (accessed on 16 April 2024).

According to Global Cancer Observatory (GLOBOCAN) data for 2022, lung cancer was the most common cancer worldwide, with nearly 2.5 million new cases, and the leading cause of cancer death in both sexes, with 1.8 million deaths, representing ≈12.5% of the global cancer incidence and ≈18.5% of all cancer deaths. Furthermore, even though lung cancer is more prevalent in males, making it the most common and deadliest cancer in this gender, it is the second cause of cancer-related deaths in females, surpassed only by breast cancer. In 2022, in Portugal, lung cancer was the third most common cancer in both males and females (≈4253 and ≈1092 new cases, respectively); it was the leading cause of cancer death in males and the third cause of cancer death in females, after breast and colorectal cancer [1,2]. Figure 1 represents an overview of the incidence and mortality of the 15 main types of cancer in each sex, corroborating the lung cancer numbers cited above.

Tobacco smoking is the most significant and well-established risk factor for lung cancer, with approximately 80% of lung cancer cases being diagnosed among smokers [3]. However, between 15 and 25% of all lung cancer cases are not associated with tobacco usage, a tendency that has increased over the past years; yet, the reasons underlying this phenomenon remain to be firmly established [4,5]. Some studies suggest the possibility of environmental exposure to tobacco smoke, occupational exposure, family history of lung cancer, hormonal factors, and lung diseases that increase predisposition, among others [5,6,7]. Interestingly, the molecular landscape of lung cancer in never-smokers is unique and without significant tobacco smoking signatures, even in cases associated with exposure to second-hand tobacco smoke [5]. The absence of specific symptoms means that lung cancer is often diagnosed at an advanced stage of the disease. In addition, these types of neoplasms usually have an aggressive behaviour. These two important factors, among others, help to explain the high mortality rate of lung cancer [8].

Currently, there are internationally accepted guidelines that were defined to ensure the best healthcare for patients afflicted with lung cancer. These guidelines address lung cancer screening, diagnosis, molecular characterization, and treatment. However, the recommended lung cancer patient path faces challenges in the real daily clinical setting and, thus, there are still several gaps in the clinical course for lung cancer patients worldwide. Here, we review the most widely accepted recommendations for the journey of a lung cancer patient, covering aspects on the ideal patient screening programmes, diagnosis, molecular characterization of tumoural samples, and patient-tailored treatment. Finally, we highlight the current daily challenges faced by the Portuguese healthcare setting in dealing with these patients, advancing potential solutions that will help to their quality of life and overall survival.

## 2. Screening

Given the non-specific symptoms of lung cancer, screening individuals at high risk of developing this type of tumour is crucial for detecting the disease in the early stage and therefore reducing the mortality attributed to this cancer type [9].

### 2.1. History of Lung Cancer Screening: What Is Currently Recommended?

Lung cancer screening (LCS) recommendations have evolved over the years. In the beginning, chest radiography was recommended for smokers or former smokers, with sensitivity in detecting tumours of approximately 1 cm in diameter [10,11]. However, death reduction using this technique was not significant and in 1980, the American Cancer Society (ACS) retracted the recommendations for the use of chest X-ray for LCS. Later, in 2013, the randomized controlled trial National Lung Screening Trials (NLST) demonstrated a mortality reduction of 20% with annual low-dose computed tomography (LDCT). The ACS changed the guidelines, recommending an annual screening with LDCT in adults aged 55–74 years who currently smoked or had a history of smoking, having stopped smoking within the last 15 years, and who had a 30 or greater pack-year history of smoking [11]. Compared to chest X-ray, LDCT has a higher specificity (93.8% compared to 73.4% for radiography), allowing for the analysis of the entire chest in a reduced time, and being more effective in the detection of small lesions (1–5 mm), with a lower exposure to unnecessary harmful radiation [10,12].

However, in 2021, the ACS updated its recommendations and extended LCS to patients aged between 50 and 80 years and with at least a 20 pack-year history of smoking. This update is supported by another large randomized controlled trial—the Nederlands-Leuvens Longkanker Screenings Onderzoek (NELSON). Therefore, the 2023 ACS guidelines are based on this last update, and it is recommended that high-risk patients perform an annual LDCT [11,13]. Table 1 summarizes the 2023 ACS guidelines for lung cancer screening. LCS recommendations for never-smokers are yet to be established.

### 2.2. Benefits and Risks of LDCT

Early detection through LCS is essential for reducing the mortality associated with the disease; this is the major benefit of using LDCT as the screening methodology [11]. However, LDCT is not free from risks; thus, the potential negative consequences associated with LCS need to be taken into consideration, and informing patients about them should be mandatory for clinicians. Radiation exposure and the risk of false-positive results are among the two major concerns related to LDCT. Naturally, a false-positive result will trigger the need to carry out additional diagnostic tests involving invasive methods to assess the possible lesions in more detail, which contributes to increasing the anxiety of the screened patient [9,14,15]. The methods for LCS are reviewed in more detail below.

## 3. Diagnosis

After a positive imaging test result, it is important to perform a histopathological analysis to confirm the neoplastic nature of the nodule and to determine the specific type of lung cancer [9]. The evaluation of the extent of the tumour—TNM stage—is also essential for a complete diagnosis. In line with this, it is key to obtain an adequate sample through biopsy and/or aspiration of bronchial fluid containing neoplastic cells. All these data are crucial for tumour classification and staging and for the therapeutic decision [16,17].

### 3.1. Histopathological Analysis

Tumour biopsy is the first method of obtaining tissue in an amount sufficient for the confirmation of a neoplastic nodule and for conducting the necessary complementary tests (immunohistochemistry, for example), which are critical for the determination of the specific type of lung cancer. Tumour biopsy has a diagnostic accuracy exceeding 88% [18]. Transthoracic needle biopsy is the most commonly used for lung cancer diagnosis, and it is performed along with CT guidance to obtain tumour tissue (CT-guided transthoracic needle biopsy) [10,12,19]. This technique is described as having a sensitivity of 93% and a specificity of 100% in detecting malignant lesions. There is still a significant (25%) complication rate associated with transthoracic needle biopsy and the most frequent complication is pneumothorax [19].

Bronchoscopy, together with cytology, are other two techniques used for lung cancer diagnosis [12]. Bronchoscopy uses a white light to identify tumours, relying on the principle of light refraction. Tumours, being less refractive, appear as black areas on bright white tissue. This technique allows for an efficient detection of tumour tissue, and a biopsy can be performed at the same time. However, it is a highly invasive diagnostic method and has some limitations, particularly in identifying pre-malignant lesions with a diameter smaller than 20 mm [12,19]. Bronchoscopy along with cytology using bronchoalveolar lavage (BAL), bronchial brushing, or conventional transbronchial needle aspiration (TBNA) are other powerful combinations for lung cancer diagnosis [20]. BAL involves bronchoscopic sampling for cytological and microbiological exams. Here, a sterile saline solution is instilled via the bronchoscope channel into the distal lung segments, thus creating a suction to recapture the saline solution containing secretions from the respiratory tract. To obtain an adequate pulmonary sampling, at least 30% of instilled volume has to be recovered [21,22]. Concomitantly, bronchial brushing is another current diagnostic tool that allows for obtaining exfoliative cytologic specimens using sheathed brushes [21]. On the other hand, needle aspiration cytology has been continuously used over the years for LCD along with bronchoscopy. It is recommended for the diagnosis of endobronchial and peripheral lesions. In this technique, a rapid on-site evaluation is important during the collection procedure to allow the technician to stop sampling once sufficient material for diagnosis has been obtained. This control allows for a reduction in complications related to bronchoscopy [22,23]. In addition, endobronchial ultrasound-guided transbronchial needle aspiration (EBUS-TBNA) is another technique which is highly recommended in patients with NSCLC who need pathological mediastinal staging [24]. Cytology can also be performed without bronchoscopy, using sputum or pleural fluid. Despite sputum cytology being a non-invasive method that helps in the early diagnosis of lung cancer, its high sensitivity is reserved for centrally located tumours. In addition, although obtaining sputum is relatively simple, it can cause some discomfort to the patient. However, despite it being a technique that was used in the past, it is no longer used nowadays [10,12,25]. On the other hand, pleural fluid cytology has a better sensitivity than sputum cytology (60–70%) and is capable of analysing the presence of malignant cells in the pleural fluid, leading to a reduced occurrence of misdiagnoses. Nonetheless, when compared to sputum collection, pleural fluid acquisition is highly invasive, and patient adherence is poor [12,26]. Table 2 summarizes the diagnostic techniques in use, with their corresponding advantages and disadvantages.

Therefore, according to its histological characteristics, lung cancer, which is a highly heterogeneous disease, can be classified into two major groups: non-small-cell lung cancer (NSCLC) and small-cell lung cancer (SCLC). These two histological types are vastly different due to being associated with specific mutational profiles, which consequently leads to distinct patient outcomes and therapeutic approaches [28]. Specifically, SCLC comprises 10–15% of all lung cancers and is related to tobacco smoking. It is poorly differentiated and is a more aggressive type with early-stage metastasis—approximately 70% of patients already exhibit metastases at the time of diagnosis. This type of lung cancer is characterized by small cells, with tiny or no cytoplasm, and the presence of necrosis [10,29]. On the other hand, NSCLC comprises approximately 85% of all lung cancer diagnoses. It can be divided into three different subtypes: adenocarcinoma, squamous-cell carcinoma, and large-cell carcinoma. Adenocarcinoma is the most common type and is more frequent among non-smoking females. Histologically, this common subtype is distinguished by larger cells and a glandular pattern [10,12,30].

Immunohistochemistry can also be required for a clear diagnosis and to distinguish SCLC from NSCLC. As of the date of the publication of this article, several neuroendocrine markers are commonly used to identify SCLC, such as insulinoma-associated protein 1 (INSM1), chromogranin A, neuron-specific enolase, neural cell adhesion molecule (NCAM, CD56), and synaptophysin. However, these markers cannot be used alone, as approximately 10% of NSCLC cases are immunoreactive for at least one of these markers. Therefore, the analysis of these neuroendocrine markers must be combined with others, like thyroid transcription factor-1 (TTF-1), which is positive in 85–90% of SCLC cases. Additionally, markers like cytokeratin (AE1/Ae3, CAM5.2) or p40 are also utilized, with p40 usually being negative in SCLC and useful for differentiating SCLC from poorly differentiated NSCLC [31].

### 3.2. Molecular Analysis

After diagnosis and tumour histological classification, NSCLC patients should undergo molecular analysis to evaluate the genetic alterations (biomarkers) that are useful for clinical management. The analysis of these mutations is conducted through tumour biopsy and several methods can be used for tumour profiling, including fluorescence in situ hybridization (FISH), next-generation sequencing (NGS), real-time polymerase chain reaction (real time-PCR), and immunohistochemistry (IHC) [32,33,34,35].

The most imperative genetic alterations to be tested are well established by the NCCN international guidelines and their testing is crucial for treatment decisions [36]. These genetic alterations include *EGFR*, *KRAS*, *BRAF*, *HER2*, and *MET* mutations/amplification, as well as *ALK*, *ROS1*, *NTRK1,2,3*, and *RET* rearrangements. At the protein level, PD-L1 evaluation is also important for therapeutic decision-making [32,34,35]. Thus, to stablish the most important targets to be considered for lung cancer treatment, the ESMO Scale for Clinical Actionability of Molecular Targets (ESCAT), which ranks the match between a mutation and a certain drug on different levels, was developed. Specifically, mutations categorized as ESCAT I are those with a high match and include *EGFR*, *BRAF*, *ALK*, and *ROS1* alterations. On the other hand, alterations such as *KRAS*, *MET*, *HER2*, *NTRK*, and *RET* are classified as ESCAT IC or II, and their testing is not yet routinely recommended. However, additional analyses are required to allow potential patients to participate in clinical trials that focus on these alterations [32,37].

Moreover, despite reflex testing not yet being universally accepted, it is already performed in some centres. This protocol is simple and quick, enabling the pathologist responsible for the case to request the test for some biomarkers as soon as the pathological diagnosis is confirmed, without the need for a formal oncologist request. Thus, the implementation of reflex testing ensures that more patients are tested, allowing for quicker decisions regarding personalized treatment options [38]. The most recent international guidelines recommend reflex biomarker testing for all patients with a diagnosis of NSCLC, independently of the stage of the disease. *EGFR* is the most important biomarker to integrate in this protocol [39].

### 3.3. TNM Classification

Lung cancer is staged according to the TNM (Tumour–Node–Metastasis) system, defined by the American Joint Committee on Cancer (AJCC)/Union for International Cancer Control (UICC). Tumour staging is performed with imaging techniques such as computed tomography (CT), positron emission tomography (PET), positron emission tomography/computed tomography (PET/CT), or magnetic resonance imaging (MRI). Depending on the type of metastasis, certain techniques may be more sensitive than others. For example, for brain metastasis detection, MRI is the best option, whereas PET/CT is preferred for the analysis of bone metastases [18,31].

### 3.4. Treatment Options

Surgery is the first treatment option for patients with early-stage NSCLC (stages I or II) [40]. Commonly, in SCLC, patients are treated with systemic therapy using cisplatin or carboplatin and in extensive-stage SCLC, NCCN guidelines recommend the combination of chemotherapy and immunotherapy targeting PD-L1 (atezolizumab or durvalumab). However, in a palliative approach, radiotherapy is also an option [18,31].

**Figure 2:**
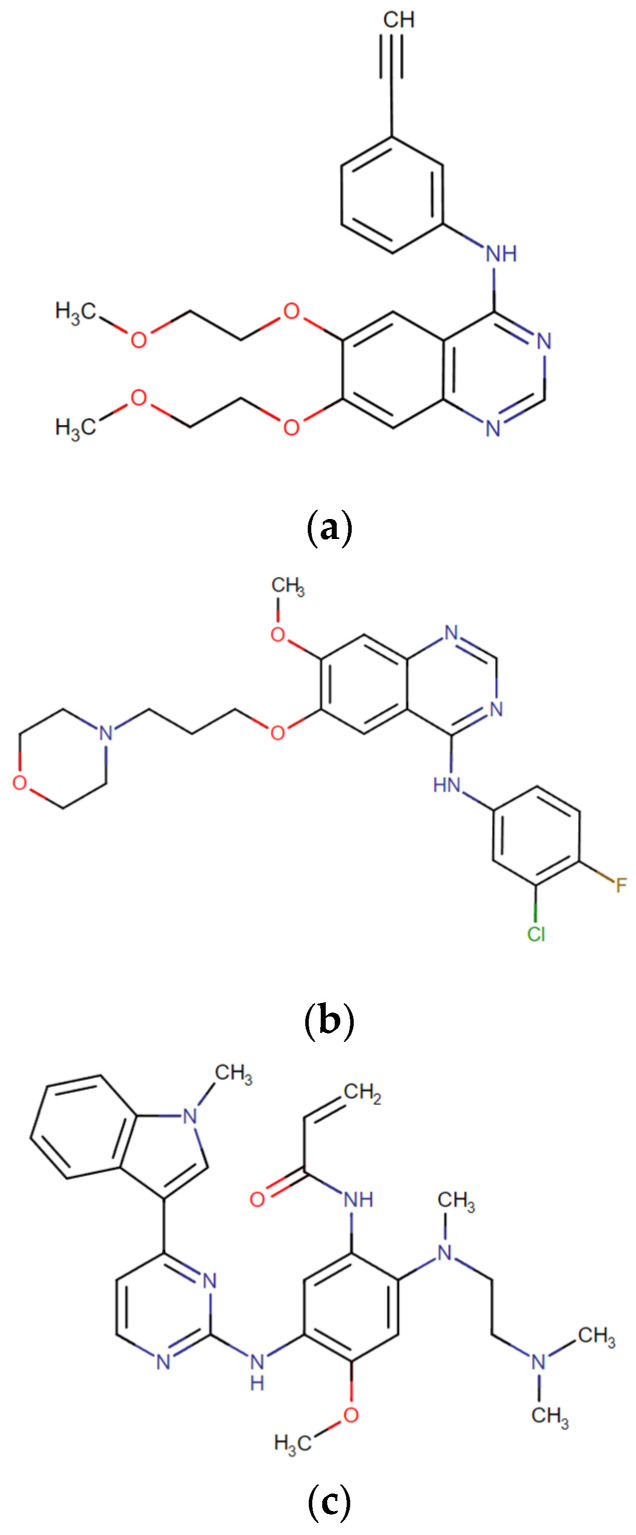
Chemical structures of (**a**) erlotinib; (**b**) gefitinib; and (**c**) osimertinib. Available online: https://go.drugbank.com/ (accessed on 23 February 2024).

For NSCLC, in the absence of oncogenic-driven mutations, the treatment approach is chemotherapy using platinum doublets combined with a third-generation cytotoxic agent (gemcitabine, vinorelbine, and taxanes). Alternatively, if there are no contraindications for immunotherapy, nivolumab may be considered along with chemotherapy when PD-L1 is overexpressed (PD-L1 > 50%) [18,36]. Considering the targeted therapies for driven mutations, TKIs are highly used in lung cancer, especially for *EGFR* mutations, namely deletions in exon 19 or point mutations in exon 21 (L858R). These mutations have a prediction response to first-generation TKIs, such as erlotinib and gefitinib [41]. However, 50–60% of patients acquire the EGFR T790M mutation, responsible for resistance to first-generation TKIs. Therefore, and according to the NCCN guidelines for 2023, osimertinib is a third-generation inhibitor recommended for this *EGFR* mutation status [35,36]. Considering *ALK* rearrangements, crizotinib is the first-line therapy approach. Nonetheless, second-generation ALK inhibitors, ceritinib and alectinib, have also been approved by the U.S. Food and Drug Administration (FDA) for patients who stop responding to the first therapeutic option [42]. Crizotinib as first- or second-line monotherapy is also approved for *ROS1* translocation. In fact, the histological profile of tumours harbouring *ROS1* or *ALK* translocation is very similar, featuring the presence of signet ring cells [43]. The *BRAF V600* mutation represents 50% of the *BRAF* mutational status in NSCLC, and it can co-exist with *KRAS* mutations. Here, a combined treatment with a BRAF and a MEK inhibitor, dabrafenib and trametinib, respectively, is recommended [18,43,44]. Finally, given the moderate frequency of tumours with high expression of PD-L1, the recommended treatment is immunotherapy using the antibodies atezolizumab or pembrolizumab [35,36,45]. Concomitantly with these main targeted therapies, there are others that have already been approved by the FDA for lung cancer treatment targeting *KRAS* (G12C mutation)*, MET* (exon 14 skipping)*, NTRK, HER2,* and *RET* fusion. In the end, testing for the most common mutational profile (PD-L1, *EGFR,* and *ALK*) before administering any targeted therapy is crucial, once patients with *EGFR* or *ALK* abnormalities are less responsive to immune checkpoint inhibitors [32,36]. Figure 2 shows the chemical structure of the three TKIs—erlotinib, gefitinib, and osimertinib—applied in the most common genetic alteration, i.e., *EGFR*. The recommended doses for these most common therapeutic approaches are 150 mg/day, 250 mg daily, and 80 mg/day for a median of 260 days or 160 mg/day for 171 days for erlotinib, gefitinib, and osimertinib, respectively [46]. Table 3 summarizes the targeted therapies approved for each biomarker in the treatment of advanced NSCLC and the respective frequencies of each biomarker recommended for testing in the present year.

### 3.5. Drug Resistance

Despite the previously cited targeted therapies improving progression-free survival and overall survival of patients with NSCLC, most of them develop drug resistance after approximately one year. Therefore, research aimed at defining the mechanisms of drug resistance at the genome, epigenome/transcriptome, and tumour microenvironment levels is important to increase therapy response [48]. Resistance to targeted therapies can be globally classified as primary (intrinsic) resistance or acquired resistance; the latter is the most common in NSCLC [49]. Specifically, intrinsic resistance is when no therapeutic effect is achieved in the initial treatment. On the other hand, acquired resistance is characterized by an initial maximal therapeutic response followed by the persistence of a sub-population of drug-resistant tumour cells, leading to disease progression [50]. Epigenetic alterations, such as DNA methylation or histone modifications, are one of the main reasons for drug resistance as they promote the escape of tumour cells to the immune system. Complementarily, changes in the proteins of tumour cells and changes in downstream signalling pathways are genetic alterations which are also highly associated with therapeutic resistance [51,52,53]. In practice, genetic alterations can be detected using several laboratory techniques, namely qPCR for *EGFR*, *BRAF*, and *KRAS* mutations, IHC or FISH for ALK and ROS rearrangements, and *MET* gene amplification. More recently, NGS has been used to search for alterations such as *MET* exon 14 Skipping, *RET*, and *NTRK1,2,3* [54]. Table 3 summarizes possible second-line targeted therapies against acquired mutations such as T790M, *KRAS^G12C^*, *MET* exon 14 skipping/amplifications which have already been FDA-approved and which aim to combat therapeutic resistance to first-line drugs.

Considering genetic modifications, in *EGFR*, the T790M mutation is a secondary acquired mutation in exon 20 of the EGFR kinase domain which leads to a substitution of methionine for threonine at position 790 [48], leading to resistance to erlotinib and gefitinib. However, the reasons why the other 40–50% of patients without this condition develop resistance to EGFR-TKIs are still not completely elucidated. Nonetheless, some mechanisms have been proposed, such as point mutations in EGFR-TK that are not responsive to TKIs, activation of alternative pathways, such as the RAS/RAF/MEK/ERK signalling pathway, and coexistence of different driver mutations like *KRAS*, *HER2*, *RET*, and *MET* [55,56]. Moreover, the *C797S* mutation has been reported to be responsible for osimertinib resistance as well as *MET*-amplification, which is described as being the most common resistance mechanism to third-generation EGFR TKIs [57,58,59]. Consequently, clinical trials were performed to evaluate combined therapy with osimertinib and a *MEK* inhibitor (NCT03392246) in advanced *EGFR*-mutant NSCLC, and another with a *c-MET* inhibitor (NCT03778229) in advanced NSCLC with *EGFR*-mutation or *MET*-amplification [60,61]. Other rare *EGFR* mutations, namely L792H, G796R, L718Q, and G724S mutations, have also been identified in research studies as promoting resistance to osimertinib [62,63].

Resistance to *ALK* inhibitors can be classified as *ALK*-dependent or *ALK*-independent resistance according to *ALK* involvement [64]. *ALK*-dependent resistance hinders the binding of the drug to the active site and approximately one-third of *ALK*-positive tumours treated with crizotinib develop some of these alterations. The first mutation of this type to be described was L1196M; however, there are others, such as G1269A, G1202R, and F1174 [48,64,65]. On the other hand, *ALK*-independent resistance is related to the aberrant activation of other kinases, such as *EGFR*, *SRC*, and *MEK*/*ERK* [66]. Considering the tyrosine kinase domain homology between *ALK* and *ROS*-1, the mechanisms of resistance in *ROS-1* are analogous to *ALK*. The most common *ROS-1* mutation that confers resistance to crizotinib is G2032R, which is structurally similar to the *ALK* G1202R mutation. Although at low frequency, other *ROS-1* mutations were identified, such as D2033N and S1986F [56,67].

In *BRAF,* some possible resistance alterations were detected in samples from NSCLC patients who experienced disease progression after treatment with *BRAF* and/or *MEK* inhibition. The most common way of developing resistance is the reactivation of the MAPK pathway. Other alterations are activating *KRAS*/*NRAS* mutations, *MEK* overexpression or mutations, mutations in *PI3K* or *AKT*, and *BRAF* amplification or alternative splicing [68,69].

Finally, anti-PD-L1 immunotherapy blocks the PD1/PD-L1 axis, enabling the recognition and killing of tumour cells by the immune system [70]. However, as in the other targeted therapies, the development of resistance to immunotherapy often occurs and the most common mechanism described in this regard is the decline of tumour antigen recognition by immune cells. Specifically, some drug resistance-related mechanisms of anti-PD-L1 immunotherapy are abnormal expression of molecules responsible for processing and presenting tumour antigens (MHC-I), the release of several immunosuppressive factors, i.e., adenylate, indoleamine 2,3-dioxygenase 1 (IDO), prostaglandin E2 (PEG2), interleukin-10 (IL-10), and transforming growth factor-β (TGF-β)-, as well as immunosuppressive cells—T regulatory cells and natural killer T (NKT) cells—and also changes in the tumour microenvironment that can promote the proliferation and release of some factors which inhibit the normal function of the immune system [70,71,72]. Therefore, considering the 30% effectiveness rate of immunotherapy in lung cancer and the development of resistance to immunotherapy, it is increasingly important to define biomarkers that help to predict therapeutic efficacy. As described before, the PD-L1 expression level is one of the most important biomarkers to be considered [70,73].

## 4. Disease Monitoring

Based on histological and molecular analysis, patients receive the most adequate therapy; therefore, they should be followed in order to monitor disease evolution and adjust the therapeutic approach in response to new possible molecular alterations. Currently, this follow-up is conducted through PET/CT scans and tumour biopsies [74]. However, the detection and analysis of cell-free DNA (ctDNA) from cancer cells, known as liquid biopsy, has proven to be extremely useful. This approach allows for the non-invasive evaluation of the tumour mutational burden through the collection of a blood sample. Compared to tumour biopsies, which only analyse a small portion of the tumour, providing a limited understanding of tumour heterogeneity, ctDNA offers an advantage by providing a better perception of all tumours, given that all cell types present in the tumour can be accessed, offering a representative overview of tumour composition. Despite the low concentrations of ctDNA in the bloodstream, advanced-stage cancer patients commonly exhibit elevated levels of ctDNA [74,75].

In fact, liquid biopsies are useful for disease monitoring but also for diagnosis. Recently, the analysis of ctDNA via NGS for the diagnosis of advanced-stage NSCLC has appeared as a promising alternative which offers several advantages over traditional tumour biopsy, namely being a less invasive technique, not requiring hospitalization, and enabling faster result delivery [76]. Complementary to ctDNA, the analysis of circulating tumour cells (CTCs) is gaining interest due to the genetic information that they can provide, as only CTCs are able to provide insights about metastases and interactions with other circulating cells [77].

## 5. Lung Cancer Research in Portugal

There are several research groups in Portugal which are focused on discovering more about lung cancer in order to understand the evolution of this disease, assess disparities between regions, and gain a better comprehension of the molecular and genetic characteristics of this tumour, trying to improve patients’ outcomes. A recent epidemiological study analysed the proportion of lung tumours associated with tobacco smoking in Portugal and the disparities between the regions. It found that the majority of lung tumours and consequent deaths are related to tobacco, whose consumption differs throughout the country [78]. Concomitantly, other Portuguese research groups recently analysed the influence of sex, socio-demographic characteristics, and tumour characteristics in lung cancer patients’ outcomes. With this study, the research group verified that the male gender and older age are associated with a poor prognosis. A late-stage diagnosis was also linked with worse survival [79]. Two other research groups have tested new therapeutic approaches, namely beta-adrenergic blockade and combination therapies using nanoparticles, to improve the therapeutic options for lung cancer patients [80,81]. Specifically, the first group evaluated the therapeutic effect of beta-blockers during immunotherapy and, although no statistically significant evidence for beta-blockers increasing immunotherapy response has been found, the modulation of the immune system with adrenergic blockage seems promising [80]. On the other hand, regarding the study on nanoparticles, its results demonstrated that anti-CD147 targeted liposomes (LUVs) carrying phenformin are effective against lung cancer cells, reducing their aggressiveness [81]. ctDNA analysis is another important area under investigation in our country. One research group demonstrated that ctDNA is useful in the detection of actionable mutations in early-stage lung cancer [82]. In addition, a recent study estimated the benefit of LDCT for LCS in high-risk patients, proving the importance of this screening [83].

Thus, all the previously cited studies prove the involvement of Portuguese research groups in the field of lung cancer, reinforcing the efforts to increase treatment options and raise awareness about the importance of implementing a lung cancer screening programme.

## 6. What Is Missing?—The Reality of a Lung Cancer Patient’s Journey in Portugal

Ideally, the journey of a lung cancer patient should follow all the previously recommended steps for early diagnosis and a personalized treatment, improving survival. Additionally, although there is no consensus on how long the assessment of a patient with suspected lung cancer should take, this should be as quick as possible. Guidelines from the College of American Pathologists, the International Association for the Study of Lung Cancer, and the Association for Molecular Pathology suggest that the turnaround time between the biopsy and the reporting of results should be about 10 days [84]. In agreement with this, the ASCO guidelines recommend a response time of less than 14 days [85].

However, in the regular clinical routine, each patient follows a different diagnosis and treatment path depending on the healthcare system [86]. Therefore, considering the more dedicated and personalized treatment of lung cancer, as in other diseases, the healthcare system needs to be more adapted to the new reality to provide the best care to patients. Considering this evolution, there are currently several gaps in the clinical process that lead to a delay in the diagnosis and treatment of patients. Considering the Portuguese context, there is no established LCS programme and the population must be urgently warned about the importance of implementing it. Moreover, despite the requirement, in Portugal, for all patients suspected of having lung cancer to receive urgent care to ensure rapid diagnosis and prompt initiation of treatment, delays and disparities between public and private hospitals still exist. In addition, the limited number of specialized radiologists is another serious aspect that requires attention.

**Figure 3:**
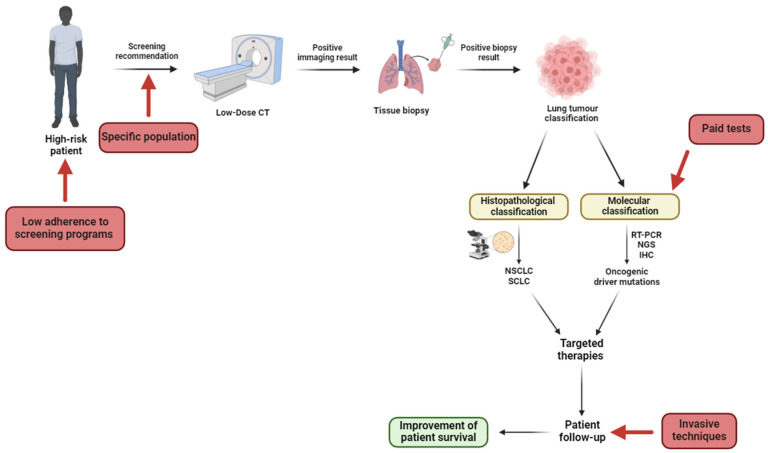
A lung cancer patient’s journey according to the recommended guidelines. The green box represents the ideal outcome if the entire pathway is followed; red boxes highlight the gaps in clinical patient management identified in Portugal. Created with BioRender.com. Available online: http://biorender.com/ (accessed on 2 February 2024).

Considering the previous brief description of the reality of Portuguese clinical management, Figure 3 illustrates a schematic representation of the recommended lung cancer patient journey, highlighting key failures in current clinical management. After that, these gaps are reviewed in detail.

Early diagnosis and proper treatment increase lung cancer patient survival by 20% [87]. However, despite the guidelines that recommend LDCT screening for high-risk patients—smokers over the age of 50 years—this has not yet been highly adopted in the clinical routine [86], which is the first major gap in lung cancer patient guidance. One of the main problems in implementing a national LCS is the limited availability of trained radiologists who can read LDCT scans and perform lung tumour biopsies [88]. This fact is an enormous limitation in Portugal, and it has become crucial to increase the number of professionals capable of carrying out this initial patient monitoring. The reduced number of centres capable of performing thoracic surgeries poses another challenge in the clinical treatment of lung cancer patients in our country. Another missing step in LCS is the definition of the target population for whom it is recommended. In fact, the majority of lung cancer cases are tobacco-related. However, the number of cases in non-smokers is increasing, even in Portugal. These cases are related to certain risk factors such as radon exposure, a family history of lung cancer, and air pollution [4]. Therefore, it would be important that LCS guidelines expand LDCT screening for non-smoker patients who may be exposed to these other risk factors. For example, people who live in the most polluted areas of Portugal or in regions with high levels of radon exposure should be considered high-risk patients eligible for LCS. Nonetheless, the low adherence to LCS programmes is a widespread problem reported by many countries attempting to implement it, and the reasons behind this low adherence vary across different countries and regions. Accordingly, strategies to implement and increase the public interest in LCS should be adapted to the specific characteristics of each population [89]. For example, in big cities, where the population has more access to technology, some strategies to raise public awareness may include direct mailing. However, in small towns and villages, where the population also tends to be older, an easier way to promote LCS is through medical centres. Social media publications are also a good way to spread the information and increase the public engagement. Concomitantly, screening recruitment should always consider the importance of informing patients about the benefits and potential risks of screening before they enrol in the programme. Thus, the patient can make a conscious choice about their participation.

Sometimes, despite the identification of a nodule in LDCT, performing a lung tumour biopsy is a challenging decision for clinicians, given that nodules are not always carcinogenic and can sometimes even disappear, depending on their nature. However, this prediction cannot be precisely made based on a single radiological image and the biopsy ends up being performed. Therefore, developing strategies such as technology with artificial intelligence to help clinicians accurately predict the carcinogenic nature of nodules is crucial in order to avoid the unnecessary submission of patients to invasive methods.

Another fact to be taken into consideration is the quality of the material obtained in biopsies. Occasionally, the quality may be poor, making the histological analysis of the tumour challenging. In addition, the quantity of sample material obtained through the biopsy may be insufficient for the necessary immunochemical and molecular tests. So, the involvement of specialized professionals in biopsy performance is essential to ensure high-quality sampling and the acquisition of as much tumour material as possible, avoiding the necessity to perform another biopsy. Moreover, despite it being highly recommended for the diagnosis/staging of mediastinal NSCLC, the number of centres offering the EBUS-TBNA technique remains low, which indicates a need to increase the accessibility of this procedure.

Tumour molecular characterization is another crucial step recommended by the guidelines for adequate lung cancer treatment. In Portugal, NGS is performed to identify targetable mutations considering a defined gene panel. However, this technique is expensive, and the context of public and private healthcare is different. In public hospitals, molecular analysis is recommended by pathologists and provided free of charge to patients. Conversely, in the private sector, NGS is paid for by the patient and despite the pathologist’s recommendation, patients must provide authorization for the analysis. Therefore, many patients choose to transfer to public hospitals for care. However, all these processes take time and can delay the patient’s treatment. Otherwise, although some health insurance providers cover the cost of molecular testing, not all do and patients who remain in the private sector do not have health insurance that covers these extra costs. Consequently, raising awareness among insurers about the importance of including these tests in their coverage packages or establishing agreements between insurers and private hospitals/laboratories would be important to enable patients to continue receiving care at their preferred hospitals and access appropriate treatment more quickly, without having to change from private to public care. In addition, regarding the public system, the effectiveness with which a suspected lung cancer patient is cared for varies from hospital to hospital, depending on whether the hospital has a “Green Lane for lung cancer” that prioritizes these patients. This protocol has a huge impact since, if it exists, the waiting time for patients to be evaluated by a professional is reduced compared to hospitals without the green lane. Therefore, it would be important that all national hospitals implement this protocol to ensure quick access to care for suspected patients.

Concomitantly, attention must be directed to the limited number of centres available for molecular testing. This problem results in occasional delays in carrying out these studies, probably due to the elevated volume of samples for NGS. Therefore, it is imperative to create additional laboratory centres able to perform such analyses, ensuring that patients have faster access to optimal therapy.

Furthermore, in NGS panels, besides the standard genes covered, it will also be important to continually update and adapt them to specific geographical regions. The inclusion of new genes would be important for research that could identify genetic signatures unique to certain populations or areas. Consequently, integrating these findings into NGS panels will improve patients’ diagnostic capabilities and increase the possibility of developing new therapies.

After treatment is chosen, patients should be followed to assess therapy response and understand if therapeutic changes are needed. Despite the proven strength and utility of liquid biopsies, they are not often the primary option in the Portuguese clinical routine. However, considering that diagnosis/follow-up techniques should be as minimally invasive as possible to ensure patient comfort, the use of liquid biopsies is a viable option that should be more frequently used in the follow-up of lung cancer patients. For example, in some cases, a new biopsy is needed to confirm the nature of metastases or to evaluate the changes in the tumour mutational burden. However, although the combined use of the two techniques may be preferable, when tissue rebiopsy is not feasible, liquid biopsy is an excellent alternative. For example, a positive liquid biopsy for a certain mutation (ex: T790M) may avoid the need for a new biopsy [90]. Nonetheless, sometimes liquid biopsy may not be the better choice, for example in cases of histological type transformation, which is a known mechanism of drug resistance.

Considering targeted therapy approaches, despite the efforts to promote and implement new treatments that could improve patient outcomes, the process of their acceptance and integration into the clinical routine is often challenging and time-consuming. Sometimes, even when a certain drug has received approval abroad, it still needs the approval of Portuguese health authorities. Furthermore, even with the accepted targeted therapies, their availability is different across different regions of Portugal, which has a decisive impact on the time it takes for patients to start treatment and, consequently, affects their outcomes.

It is known that clinical management varies across countries and the key failures and challenges in this field may be different between them. However, despite the challenges in the journey of a lung cancer patient identified above which are specific to the Portuguse context, some of them can be applied all over the world. Difficulties in implementing an LCS programme and the low number of trained radiologists able to perform lung tumour biopsies are the two most common issues reported worldwide [88]. Nonetheless, gaps in molecular testing, how quickly a patient suspected of having lung cancer is treated, and the decision to perform a biopsy or not can also be cross-country challenges. Consequently, besides improving lung patient care in Portugal, this review article is also a guide for foreign healthcare systems, alerting them to possible gaps in these patients’ follow-up and treatment and helping them identify similar problems in the field of lung cancer care and how they can improve them, thus increasing lung cancer patient outcomes.

## 7. Conclusions

A lung cancer patient should follow an approved and recommended clinical pathway that ensures the best treatment and care. In this journey, screening and tumour biopsy should be performed by a specialized radiologist, and a proper histological analysis for lung cancer type identification must be conducted by a specialized pathologist. In NSCLC, a subsequent molecular evaluation is essential for the identification of possible mutations that could be important for targeted therapies. All these steps, accompanied by a proper disease follow-up for possible therapeutic adjustments, create the ideal lung cancer patient journey. However, considering the Portuguese context, we identified some gaps in daily clinical management that may influence patient healthcare. The main gaps identified were population selection for LC screening, the number of trained radiologists, and the logistics behind the molecular testing. Therefore, new approaches to overcome these problems must be urgently determined. Specifically, creating and increasing adherence to a national LCS programme, not only for smokers and ex-smokers, but also for never-smokers who also have a high risk of lung cancer development, is an urgent matter. Additionally, creating agreements with health insurance providers to cover the cost of molecular tests for patients in the private sector would be an important step towards ensuring that all patients receive prompt and appropriate treatment in any hospital. Therefore, despite Portugal’s evolved healthcare system, the previous points are some of the gaps that must be revised and changed. In fact, although these gaps may seem minor, they play a crucial role in enhancing the quality of life and increasing the survival rates of patients.

## Figures and Tables

**Table 1:** American Cancer Society (ACS) guidelines for lung cancer screening as of 2023 [11].

Guidelines for Lung Cancer Screening 2023	
Age	50–80 years
Smoking status	Patients who currently smoke or have a history of smoking (number of years since quitting smoking is not considered)
Smoking history	≥20 pack-year history
Recommended screening test	Annual screening with LDCT
Exclusion criteria	Individuals with comorbid conditions that limit life expectancy; Individuals who do not want to be treated after a positive screening test
Decision-making	It is recommended to have a decision-making discussion with a health professional about the benefits and risks of LCS; Current smokers should be advised to stop smoking.

LDCT—low-dose computed tomography; LCS—lung cancer screening.

**Table 2:** Advantages and disadvantages of the main diagnostic approaches to lung cancer detection [12,19,20,21,22,23,27].

Diagnostic Techniques	Advantages	Disadvantages
Biopsy	High sensitivity; Evaluation of lung pleura, mediastinum, and lung parenchyma	Highly invasive; Risk of pneumothorax
Bronchoscopy	Lower risk of complications	Highly invasive; Lower sensitivity to small lesions; Less efficacy for peripheral pulmonary lesions
EBUS-TBNA	Highly specific and sensitive in mediastinal lesions	Low sensitivity in detecting micrometastases
Cytology Bronchoalveolar lavage	Minimally invasive	Low accuracy in peripheral diagnosis; There is not a standardized protocol; Quality of sample is affected by volume returned
Bronchial brushing	Good complement to biopsy results; Cost-effective strategy for diagnosis of endobronchial lung cancer	Bleeding
Needle aspiration	Safe technique; Risk of complications <1%	Difficulty in needle handling; Trouble in achieving a rapid on-site evaluation
Pleural fluid	Evaluation of all malignant cells in pleural fluid (reduced misdiagnosis)	Invasive technique

**Table 3:** Biomarkers recommended for testing, their frequency, and targeted therapy for NSCLC [18,32,45,47].

Biomarker	Frequency (%)	Approved Drug(s)
*EGFR*	10–15% (50–60% Asian)	Erlotinib; gefitinib; osimertinib (T790M mutation)
*ALK*	5%	Crizotinib; ceritinib; alectinib; brigatinib
*BRAF*	2%	BRAF/MEK inhibitor (dabrafenib/trametinib)
*ROS1*	1–2%	Crizotinib
*RET*	1–2%	Selpercatinib; pralsetinib
*MET*	3%	Capmatinib; tepotinib
*NTRX*	0.23–3%	Larotrectinib, entrectinib
*KRAS^G12C^*	12%	Sotorasib
*HER2*	2–5%	Trastuzumab deruxtecan
PD-L1	23–28%	Atezolizumab; pembrolizumab

## Data Availability

Not applicable.
